# Supplementary material for: The impact of cognitive impairment and low muscle mass on all-cause mortality among older adults in China: An empirical analysis based on CLHLS cohort data
Source: PLoS One. 2026 Mar 5;21(3):e0343609. doi: 10.1371/journal.pone.0343609 (PMC12962499; doi:10.1371/journal.pone.0343609)
Supplement: S1 File — (DOCX) [file pone.0343609.s001.docx]

Supplementary Material

**S1 Table.** VIF results of confounders included in the multivariate regression models.

| Confounders | VIF results |
| --- | --- |
| Age | 1.126 |
| Residence | 1.053 |
| Gender | 1.256 |
| Marriage | 1.060 |
| Education | 1.121 |
| Average household income | 1.030 |
| BMI | 1.023 |
| Physical activity | 1.080 |
| Drinking | 1.071 |
| Smoking | 1.094 |
| Heart disease | 1.038 |
| Hypertension | 1.049 |
| Diabetes | 1.033 |
| Stroke | 1.027 |
| BADL disablity | 1.089 |
| IADL disablity | 1.191 |
| Respiratory disease | 1.008 |
| Arthritis | 1.013 |
| Cancer | 1.001 |
| Sensory impairment | 1.094 |

**S2 Table.** Adjusted HRs of CI orLMM status and all-causes mortality, excluding the participants with a previous episode of stroke.

|  | **CI-/LMM-** | **CI-/LMM+** | **CI+/LMM-** | **CI+/LMM+** | ***p* for trend** |
| --- | --- | --- | --- | --- | --- |
|  | HR | HR (95% CI) | HR (95% CI) | HR (95% CI) |  |
| Model 1 | 1 (ref) | 1.77 (1.54–2.04) | 3.43 (2.66–4.44) | 5.74 (4.93–6.67) | <0.001 |
| Model 2 | 1 (ref) | 1.39 (1.20–1.60) | 2.37 (1.83–3.07) | 3.70 (3.15–4.36) | <0.001 |
| Model 3 | 1 (ref) | 1.23 (1.05–1.45) | 2.20 (1.69–2.86) | 3.08 (2.56–3.70) | <0.001 |
| Model 4 | 1 (ref) | 1.20 (1.02–1.42) | 1.68 (1.29–2.20) | 2.38 (1.97–2.88) | <0.001 |

Abbreviations: CI,cognitive impairment; LMM,low muscle mass; BMI, body mass index; HR, hazard ratio; ref,reference.

Model 1:unadjusted.

Model 2:adjusted for age,residence, gender, marriage, education, average household income.

Model 3:adjusted for age,residence, gender, marriage, education, average household income, physical activity,drinking, smoking, BMI.

Model 4:adjusted for age,residence, gender, marriage, education, average household income, physical activity, drinking, smoking, BMI, heart disease, hypertension, diabetes, BADL disablity, IADL disablity, respiratory diseases, arthritis, cancer, sensory impairment.

**S3 Table.** Adjusted HRs of CI or LMM status and all-causes mortality, excluding the participants with a previous episode of heart disease.

|  | **CI-/LMM-** | **CI-/LMM+** | **CI+/LMM-** | **CI+/LMM+** | ***p* for trend** |
| --- | --- | --- | --- | --- | --- |
|  | HR | HR (95% CI) | HR (95% CI) | HR (95% CI) |  |
| Model 1 | 1 (ref) | 1.87 (1.62–2.17) | 3.84 (2.97–4.96) | 5.85 (5.00–6.84) | <0.001 |
| Model 2 | 1 (ref) | 1.47 (1.27–1.71) | 2.60 (2.00–3.37) | 3.87 (3.27–4.58) | <0.001 |
| Model 3 | 1 (ref) | 1.34 (1.13–1.59) | 2.44 (1.88–3.17) | 3.32 (2.75–4.01) | <0.001 |
| Model 4 | 1 (ref) | 1.31 (1.11–1.56) | 1.82 (1.39–2.38) | 2.56 (2.10–3.11) | <0.001 |

Abbreviations: CI,cognitive impairment; LMM,low muscle mass; BMI, body mass index; HR, hazard ratio; ref,reference.

Model 1:unadjusted.

Model 2:adjusted for age,residence, gender, marriage, education, average household income.

Model 3:adjusted for age,residence, gender, marriage, education, average household income, physical activity,drinking, smoking, BMI.

Model 4:adjusted for age,residence, gender, marriage, education, average household income, physical activity, drinking, smoking, BMI, hypertension, diabetes, stroke, BADL disablity, IADL disablity, respiratory diseases, arthritis, cancer, sensory impairment.

**S4 Table.** Adjusted HRs of CI or LMM status and all-causes mortality, excluding participants who had died within 2 years of follow-up.

|  | **CI-/LMM-** | **CI-/LMM+** | **CI+/LMM-** | **CI+/LMM+** | ***p* for trend** |
| --- | --- | --- | --- | --- | --- |
|  | HR | HR (95% CI) | HR (95% CI) | HR (95% CI) |  |
| Model 1 | 1 (ref) | 1.75 (1.50–2.03) | 4.13 (3.19–5.35) | 5.03 (4.24–5.97) | <0.001 |
| Model 2 | 1 (ref) | 1.44 (1.23–1.68) | 3.12 (2.40–4.05) | 3.61 (3.01–4.33) | <0.001 |
| Model 3 | 1 (ref) | 1.33 (1.12–1.59) | 2.96 (2.28–3.85) | 3.16 (2.57–3.87) | <0.001 |
| Model 4 | 1 (ref) | 1.31 (1.09–1.56) | 2.51 (1.92–3.29) | 2.70 (2.18–3.34) | <0.001 |

Abbreviations: CI,cognitive impairment; LMM,low muscle mass; BMI, body mass index; HR, hazard ratio; ref,reference.

Model 1:unadjusted.

Model 2:adjusted for age,residence, gender, marriage, education, average household income.

Model 3:adjusted for age,residence, gender, marriage, education, average household income, physical activity,drinking, smoking, BMI.

Model 4:adjusted for age,residence, gender, marriage, education, average household income, physical activity, drinking, smoking, BMI, heart disease, hypertension, diabetes, stroke, BADL disablity, IADL disablity, respiratory diseases, arthritis, cancer, sensory impairment.
