# Supplementary material for: The impact of cognitive impairment and low muscle mass on all-cause mortality among older adults in China: An empirical analysis based on CLHLS cohort data
Source: PLoS One. 2026 Mar 5;21(3):e0343609. doi: 10.1371/journal.pone.0343609 (PMC12962499; doi:10.1371/journal.pone.0343609)
Supplement: Attachment — Submitted filename: Response to Reviewers.docx [file pone.0343609.s004.docx]

Dear Editors and Reviewers,

We are profoundly grateful for the opportunity to resubmit our work. We wish to thank the Editor for their time and the reviewers for providing such meticulous and valuable feedback. Your professional expertise and rigorous attitude have inspired our team significantly, and we feel that our research has been greatly enhanced by your guidance. We sincerely apologize for any shortcomings in the previous version and are deeply moved by the time you spent helping us refine our study. We have revised the paper to the best of our ability and remain open to any further suggestions you may have.

**Responses to Reviewer 1**

**Comment #1:** Although an excellent paper, Reference #4 does not provide support for the statement that low muscle mass is highly prevalent. Please provide appropriate evidence.

**Response:** We thank the reviewer for this important observation. We carefully re-evaluated Reference #4 and agree that it does not directly support the statement regarding the high prevalence of low muscle mass. We have therefore revised the relevant sentences and added population-based epidemiological evidence demonstrating the prevalence of low muscle mass among older adults. Specifically, we incorporated recent large-scale studies based on the CLHLS and other population-based cohorts to provide appropriate empirical support **(revised Introduction, Lines 53–55, Reference: Associations of Chinese-modified MIND diet with low muscle mass and physical performance among old adults in China: findings from the CLHLS 2018 national survey. )**

**Comment #2:** The term “1.57-fold” is not appropriate; a 57% increase in risk would be more accurate.

**Response:** We appreciate this helpful clarification. We have revised the wording accordingly, replacing “1.57-fold” with “a 57% increase in the risk of all-cause mortality,” which more accurately reflects the magnitude of the association **(revised Introduction, Line 70)**

**Comment #3:** Please cite at least one population-based observational study to support this point.

**Response:** We agree with the reviewer’s suggestion. We have added a population-based observational study examining the association between cognitive impairment and low muscle mass in older adults to support this statement. The corresponding citation has been included in the revised Introduction **(Lines 72–76, Reference: The relationship between skeletal muscle mass and cognitive impairment in older adults: a longitudinal study based on CLHLS.)**

**Comment #4:** Reference #10 appears to be incomplete.

**Response:** Thank you for pointing this out. We have carefully checked Reference #10 and corrected the incomplete information. The reference list has been updated to include the full bibliographic details **(Lines 476–478, Reference: 12. Yi Z. Introduction to the Chinese Longitudinal Healthy Longevity Survey (CLHLS). In: Yi Z, Poston DL, Vlosky DA, Gu D, editors. Healthy Longevity in China: Demographic, Socioeconomic, and Psychological Dimensions. Dordrecht: Springer; 2008. p. 23–38.)**

**Comment #5:** You report that 110 participants were lost to follow-up, but this is not clear in Figure 1. Please clarify.

**Response:** We appreciate this comment. We have clarified the handling of participants lost to follow-up in both the figure and the text. Specifically, Figure 1 has been revised to explicitly indicate “Lost to follow-up (n = 110),” and the Methods section now clearly states that these participants were excluded prior to the formation of the final analytical sample **(revised Methods, Lines 108; Lines 154-157)**

**Comment #6:** The relationship between education categories used for cognitive impairment cutoffs and those presented in Table 1 is unclear.

**Response:** Thank you for highlighting this issue. To improve clarity, we have added a brief explanatory sentence in the Methods section specifying how educational categories correspond across the manuscript. We now explicitly state that “illiterate” refers to 0 years of schooling, “primary school” corresponds to 1–6 years of schooling, and “secondary school or above” refers to more than 6 years of schooling. This clarification aligns the education-specific MMSE cutoff points with the categories shown in Table 1 **(revised Methods, Lines 118–122; Lines 164–165)**

**Comment #7:** The source of the prediction equation is unclear, and Reference #15 does not report any prediction equations.

**Response:** We thank the reviewer for identifying this error. We carefully rechecked the source of the appendicular skeletal muscle mass prediction equation and confirmed that it was incorrectly attributed. We have now corrected the citation by replacing Reference #15 with the original study that developed and validated the anthropometric prediction equation. The corresponding text and reference list have been revised accordingly **(revised Methods, Lines 128–131)**

**Comment #8:** There is an inconsistency between the text and Table 4 regarding which group has the highest mortality risk among participants under 80 years of age.

**Response:** We apologize for this oversight and thank the reviewer for bringing it to our attention. We have corrected the text in the Results section to ensure consistency with Table 4. The revised text now accurately states that among participants aged <80 years, the CI+/LMM+ group exhibited the highest mortality risk (HR = 3.66). All related descriptions have been carefully checked for consistency **(revised Results and Table 4, Lines 307–309)**

**Comment #9:** The statement regarding consistent findings may be simplistic, as the same cohort (CLHLS) was used. Consider comparison with other populations.

**Response:** We thank the reviewer for this thoughtful comment. We agree that the age-specific finding should be interpreted with appropriate caution. In the revised Discussion, we have clarified that the evidence regarding stronger associations among individuals younger than 80 years is derived from previous longitudinal analyses conducted within the same CLHLS cohort, rather than from independent external populations. Accordingly, we have framed this observation as supportive background evidence suggesting potential age-related heterogeneity, rather than as a universally established finding. Our study therefore serves as an independent longitudinal validation of this age-related pattern within a large nationally representative cohort **(revised Discussion, Lines394–397)**

We are deeply grateful to the reviewers and the editorial team for the insightful guidance provided throughout the review process. Your expertise has been instrumental in enhancing the quality of our work. We sincerely appreciate your continued consideration of our revised manuscript and look forward to your feedback.

Sincerely,

Jiefen Ou

Corresponding author: Jiefen Ou

Email: Ojf2854529676@163.com
